# Tuberculosis associated immune reconstitution inflammatory syndrome in patients infected with HIV: meningitis a potentially life threatening manifestation

**DOI:** 10.1186/1742-6405-9-17

**Published:** 2012-05-23

**Authors:** Upasna Agarwal, Amitabh Kumar, Digamber Behera, Martyn A French, Patricia Price

**Affiliations:** 1 LRS Institute of Tuberculosis and Respiratory Diseases, Sri Aurobindo Marg, near Qutab Minar, New Delhi, India, 110 030; 2 School of Pathology and Laboratory Medicine, University of Western Australia, and Department of Clinical Immunology and Immunogenetics, Royal Perth Hospital, Perth, Australia

**Keywords:** Tuberculosis, HIV, IRIS, Meningitis

## Abstract

**Background:**

Tuberculosis (TB) is the most common co infection in HIV-infected persons in India, requiring concomitant administration of anti TB and antiretroviral therapies. Paradoxical worsening of tuberculosis after anti-retroviral therapy (ART) initiation is frequently seen.

**Objective:**

To study the frequency, clinical presentation and outcome of paradoxical tuberculosis associated immune reconstitution inflammatory syndrome (TB-IRIS) in HIV infected patients in a TB hospital in North India.

**Design:**

A retrospective chart review of HIV-infected TB patients on anti-tubercular treatment (ATT) at time of ART initiation over a 3 year period. Medical records were reviewed for clinical manifestations and outcome in patients who developed TB-IRIS.

**Results:**

514 HIV-infected patients were enrolled between January 2006 and December 2008. Thirteen (12.6%) of 103 patients who had received ART and ATT simultaneously developed paradoxical TB-IRIS. Clinical presentations of paradoxical TB-IRIS included new lymphadenopathy (n = 3), increase in size of existing lymphadenopathy (n = 3), worsening of existing pulmonary lesions (n = 2), appearance of new pleural effusion (n = 1) and prolonged high grade fever (n = 2). Four patients developed new tubercular meningitis as manifestation of TB-IRIS. Our cases developed TB-IRIS a median of 15 days after starting ART (IQR 15–36). TB-IRIS patients were older (> 35 years) than those with no IRIS (P = 0.03), but were not distinguishable by CD4 T-cell count, duration of ATT before ART or the outcome of TB treatment. Eight (62%) patients had a complete recovery while 5 (38%) patients with TB-IRIS died, of which majority (n = 3) had meningitis.

**Conclusions:**

Paradoxical TB-IRIS is a frequent problem during concomitant ATT and ART in HIV-TB co infected patients in north India. Meningitis is a potentially life threatening manifestation of TB-IRIS.

## Introduction

Tuberculosis (TB) is the most common co-infection in HIV-infected persons in high burden countries like India [1]. Many HIV patients consequently require concomitant anti-tubercular treatment (ATT) and antiretroviral therapy (ART). Paradoxical worsening of TB after ART initiation is termed as TB associated immune reconstitution inflammatory syndrome (TB-IRIS) as these exaggerated inflammatory responses are attributed to immune recovery. TB-IRIS is characterized by either deterioration of existing lesions (paradoxical TB-IRIS) or the appearance of new disease associated with previously subclinical infection (unmasking TB-IRIS) after ART initiation [2]. These conditions probably reflect rapid restoration of pathogen-specific immune responses with ART.

Paradoxical TB-IRIS has varied clinical presentations. In some cases, it may present as mild lymph node inflammation alone, while in others it may manifest as potentially life threatening complications like meningitis or acute respiratory distress syndrome [3]. In a clinical setting, paradoxical TB-IRIS needs to be differentiated from other complications of ART and HIV infection like drug toxicities, development of new opportunistic infection, failure of treatment and possible non-adherence to treatment. In this paper, we have evaluated the frequency, clinical presentation and outcome of paradoxical TB-IRIS in patients attending an HIV clinic in a referral TB hospital in North India.

## Methods

This study was a retrospective analysis of prospectively collected data obtained in a structured format from patients with HIV infection and TB disease who were on ATT at time of ART initiation. Detailed case notes were available until the end of ATT, so occurrence of IRIS episode and the time of IRIS development could be accurately analysed.

### Patient selection

We reviewed structured patient charts for 103 adult HIV-infected patients, who had active TB and were treated for tuberculosis and HIV disease at the HIV clinic of LRS Institute of TB and Respiratory Diseases, New Delhi, between January 2006 and December 2008.

All patients included for this analysis were adults, had active TB at entry to HIV care at the clinic and were started on ATT as soon as TB was diagnosed. None of them were on antiretroviral therapy at time of TB diagnosis. Baseline (pre-ART) CD4 T-cell counts were done and combination antiretroviral therapy was initiated during ATT, as soon as patient stabilized on ATT, as per the National ART guidelines [4]. Data till outcome of TB treatment was available. Follow-up period till completion of TB treatment was taken for the analysis as TB-IRIS occurs during concomitant TB and ART. End points were defined as occurrence of TB-IRIS, completion of ATT or death/default while on TB treatment. HIV-TB cases who died or defaulted while on ATT alone and could not be initiated on ART were excluded. IRIS episodes not associated with *M. tuberculosis* infection were not considered in this analysis.

### Diagnosis and treatment of TB

TB was diagnosed as per existing WHO and Revised National Tuberculosis Control Program (RNTCP) [5] guidelines by identification of typical clinical features, isolation of acid fast bacilli (AFB) from a clinical specimen wherever possible, radiological findings and decision to treat for TB. TB was classified either as smear-positive pulmonary TB, smear-negative pulmonary TB, or extra-pulmonary TB. Investigations available for patients included sputum smear microscopy for AFB, mycobacterial culture and drug sensitivity, chest radiology, abdominal ultrasonography, fine needle aspiration for cytology, AFB smear / culture and computed tomography wherever indicated. ATT comprised standard short course regimens available under the RNTCP which consist of rifampicin, isoniazid, pyrazinamide and ethambutol with additional streptomycin in retreatment cases. Outcomes of TB treatment also had been recorded in accordance to the RNTCP guidelines as Cured, Treatment completed, Died, Failure and Defaulted [5].

### Diagnosis of HIV and antiretroviral therapy

HIV infection was diagnosed using three antigenically different rapid kits as per the national HIV testing policy. ART regimens followed WHO and National ART Program guidelines [4] and comprised efavirenz in combination with either zidovudine or stavudine and lamivudine; the dose of efavirenz for all adult HIV infected patients on ATT co-prescription being 600 mg once a day, in accordance to the national guidelines. CD4 cell counts were determined by flow-cytometry technique using Facs Count machine with Facscount™ reagents (Becton Dickinson, USA). As per Program guidelines, CD4 T cell counts were determined before treatment initiation and every six months thereafter. Plasma HIV viral load testing is not available under the Program for patients on first line ART regimens.

### Diagnosis and management of paradoxical TB-IRIS

The paradoxical TB-IRIS cases documented in the records were diagnosed at the time as an initial symptomatic improvement during ATT followed by an exacerbation of symptoms, signs or radiological manifestations of TB after initiation of ART, which was not due to another opportunistic infection, drug adverse effect or ineffective TB treatment [6]. TB–IRIS cases had been diagnosed at the time of presentation of symptoms by a concurrence of opinion of two trained HIV physicians, after exclusion of other causes. Sputum samples of patients who developed pulmonary manifestations of TB-IRIS were sent for AFB culture and sensitivity testing to exclude drug resistant TB. Cases manifesting CNS symptoms had undergone cerebrospinal fluid examination – cytology, biochemical analysis, India ink preparation and smear/culture for AFB. Contrast enhanced CT scans of the brain though not routinely done, were available for all 4 cases with CNS symptoms. TB-IRIS episodes were managed with non-steroidal anti- inflammatory drugs (NSAIDS) and/or steroids, in addition to ATT and ART. Serious cases received in-patient care.

At time of this analysis, the paradoxical TB-IRIS cases documented in the records were also compared to the International Network for the Study of HIV-associated IRIS (INSHI) case definition for paradoxical TB-associated IRIS for resource limited settings published in 2008 [2] . Ten of 13 cases conformed to the criteria recommended by INSHI for paradoxical TB-IRIS. However all criteria could not be met in 3 cases. In one case, the time of occurrence of TB-IRIS was nearly four months as compared to three months specified in the INSHI definitions and in the other two, high grade protracted fever was the only manifestation of IRIS.

This study was approved by the Institutional Review Board of LRS Institute of TB and Respiratory Diseases, New Delhi, an autonomous institute under the Ministry of Health and Family Welfare, GOI.

### Data analysis

Continuous data are presented as median and interquartile range (due to extreme values). Due to small sample size, the continuous variables were compared using Mann–Whitney non parametric statistics. Proportions of categorical variables were compared using chi – squared test (univariate analysis). Yates correction was applied for cells with expected frequency less than 5. All tests were 2-tailed and p <0.05 was considered statistically significant.

## Results

A total of 514 HIV-infected patients were registered at the HIV clinic of LRS Institute of TB and Respiratory Diseases, New Delhi between January 2006 and December 2008. Of these, 103 patients had received ART and ATT simultaneously and met our inclusion criteria.

Thirteen patients (12.6%) developed worsening of pre-existing disease and were considered to have experienced paradoxical TB-IRIS. Details of patients developing paradoxical TB-IRIS are summarised in Table 1. Paradoxical IRIS manifested as new lymphadenopathy in 3 (23%), increase in size of existing lymphadenopathy in 3 (23%), worsening of existing pulmonary lesions in 2 (15%), and new pleural effusion in 1(8%) of the TB-IRIS cases. Four (31%) patients developed tuberculous meningitis after initiation of ART. Two of the four patients who developed meningitis presented with severe headache and vomiting.The other two presented with altered sensorium, one of whom also developed fever. Neck rigidity was present in all four cases. Cerebrospinal fluid examination was done in all four cases. CSF examination revealed lymphocytosis with elevated protein and reduced glucose levels. None of the four cases showed any fungal growth on India Ink staining of the CSF. In the two cases where CSF cultures were done, cultures for AFB and fungal elements were negative. Available CT scans (head) showed features suggestive of mild hydrocephalous in one, basal exudates in one and reported no abnormality in 2 cases. None of the CT scans revealed any space occupying lesion. In 2 (15%) patients, a recurrence of fever was the only manifestation of IRIS. Fever was continuous, high grade (>39.4° C) and lasted for 1 and 3 weeks. No other cause for this fever was found. Both cases were managed by NSAIDS with continued ATT and ART.

**Table 1:** Details of patients developing paradoxical TB-IRIS

**Patient**	**Age (yrs)**	**Gender**	**Baseline CD4 T-cell count (cells/μl)**	**Site of TB at initial diagnosis**	**Time between ATT & ART initiation (days)**	**Clinical/radiological manifestations of paradoxical TB-IRIS**	**Interval b/w ART and IRIS appearance (days)**	**Hospital Admission**	**Treatment given for IRIS**	**Outcome of IRIS**
1	26	Male	110	Pulmonary TB with mediastinal lymphadenopathy	18	Increase in size of mediastinal lymph nodes, new pleural effusion	14	No	Steroids, NSAIDS	Recovered
2	45	Male	6	Bilateral pulmonary TB	127	Painful new abdominal lymphadenopathy (Baseline USG abdomen was normal)	15	Yes	Steroids	Recovered
3	39	Male	106	Mediastinal lymphadenopathy	176	Severe headache, neck rigidity, TB meningitis	17	Yes	Steroids	Recovered
4	39	Male	41	Miliary TB with pleural effusion and hilar and cervical lymphadenopathy	14	Altered sensorium, neck rigidity, TB meningitis	30	Yes	Steroids	Died
5	32	Male	237	Cervical lymphadenopathy	71	Increase in size of cervical lymph node	8	No	Surgical drainage	Recovered
6	35	Male	106	Pulmonary TB	25	Recurrence of fever (high grade, prolonged)	7	No	NSAIDS	Recovered
7	38	Male	14	Bilateral pulmonary TB with empyema and abdominal lymphadenopathy	37	Increased bilateral pulmonary infiltrates	39	Yes	Steroids	Died
8	44	Male	167	Cervical lymphadenopathy	22	Increase in size of existing cervical lymph node, appearance of new cervical lymph node	6	No	Steroids	Recovered
9	51	Male	62	Pulmonary TB with abdominal lymphadenopathy	29	New inguinal lymph node	48	No	NSAIDS	Recovered
10	46	Male	181	Miliary TB	26	Severe headache, vomiting, TB meningitis	36	Yes	Steroids	Died
11	30	Male	74	Extensive bilateral pulmonary TB	27	Fever, altered sensorium, TB Meningitis	120	Yes	Steroids	Died
12	50	Male	56	Mediastinal lymphadenopathy	22	Recurrence of fever (high grade, prolonged)	12	No	NSAIDS	Recovered
13	38	Male	55	Pulmonary TB with pleural effusion	52	Increased bilateral pulmonary infiltrates	14	Yes	Steroids	Died

Six patients (47%) received out-patient care and seven (54%) required hospitalization. Nine patients (69%) required steroids as they had severe disease manifestations including meningitis, worsening of extensive pulmonary disease and compressive lymphadenopathy. Three patients were prescribed solely NSAIDS. One required surgical drainage of a lymph node abscess.

Eight (62%) of the 13 patients with paradoxical TB-IRIS had a complete recovery while 5 (38%) patients died. Deaths were attributable to meningitis in 3 patients and extensive worsening of bilateral pulmonary infiltrates in the other 2. All five had been hospitalized and started on steroids. The mean CD4 T cell count for the five cases that died was 73 cells/μl (median 25, IQR 41–74). The means of CD4 cell counts of the three meningitis patients and the other two cases that died were 99 cells/μl and 34 cells/μl respectively.

Demographic and clinical factors associated with IRIS in earlier studies were analysed in patients with and without IRIS (Table 2). No differences in baseline CD4 T-cell count or CD4 T-cell recovery on ART were evident in the two groups. TB-IRIS patients were a little older (> 35 years) than those with no IRIS (P = 0.03). They had received ATT for a shorter interval before ART initiation (27 days vs. 42 days) but this difference was not statistically significant. The outcome of treatment of TB was available in all the cases (Table 2). The success of TB treatment (cured and treatment completed cases) was similar in patients who developed TB-IRIS and those who did not. The proportion of deaths was higher in patients who developed TB-IRIS, however this difference was not found to be statistically significant (P = 0.08).

**Table 2:** Comparison of patient characteristics and outcome of TB treatment in cases with and without paradoxical TB –IRIS (univariate analysis)

	**Cases with TB-IRIS (n = 13)**	**Cases without TB-IRIS (n = 90)**	**p value**
**Patient characteristics**			
Age, in years	39 [35–45]*	32 [28–39]*	0.03
Age > 35 years, n (%)	9 (69%)	30 (33%)	0.03
Baseline CD4 T-cell count (cells/μl)	74 [55–110]*	84 [46–142]*	0.52
CD4 T cell count at six months on ART (cells/μl)	142[192–467]* (n = 7)	156 [176–423]* (n = 66)	0.85
Male gender, n (%)	13 (100%)	76 (84%)	0.27
Disseminated & extra pulmonary TB, n (%)	10 (77%)	57(63%)	0.54
Interval between ATT and ART initiation, in days	27 [22–52]*	42 [25–97]*	0.30
Interval between ART initiation and appearance of IRIS, in days	15 [12–36]*	Not applicable	
**Outcome of TB treatment**			
Successfully treated, n (%) (Bacteriologically Cured & TB Treatment Completed cases)	8 (61.5%)	70 (77.8%)	0.35
Treatment failures, n (%) (Bacteriologically positive at five or more months of TB treatment)	0	2 (2.2%)	0.59
Died, n (%) (Death during course of TB treatment)	5 (38.5%)	13 (14.4%)	0.08
Defaulted, n (%) (TB treatment discontinued for 2 or more months consecutively after initiation)	0	5 (5.6%)	0.87

*median [Inter quartile range].

## Discussion

Paradoxical TB-IRIS is a clinical entity frequently encountered when ART is initiated in HIV and TB co-infected patients. Understanding of this phenomenon is particularly relevant for HIV clinics in South East Asia and Africa where many patients with active TB enter ART programs [1,7].

Retrospective and prospective studies have reported that 7- 43% of patients with HIV-TB co-infection develop IRIS [3]. The frequency of TB-IRIS among patients starting ART during ATT in our series (12.6%) is within this range. Our cases developed IRIS a median of 15 days after starting ART (IQR 15–36) (Table 2), which is similar to that reported by other authors. In most patients, TB-IRIS is seen to develop within the first 2–6 weeks [3,7-10], though symptoms can occur as early as five days after starting ART. Case reports describing development of TB-IRIS after the first three months of ART initiation are infrequent [11].

Baseline CD4 T cell counts or CD4 T cell recovery after six months of ART were not different between patients who developed TB-IRIS and those who did not. Earlier studies from South India [9] and Thailand [12] report similar findings. However, there are cohort studies from South Africa [7,13] which report a definite association of development of TB-IRIS with low baseline CD4 counts. The reason for this difference is not clear. It may be a reflection of the overall low CD4 counts of the cohort of HIV-TB cases in this analysis.

In our study from North India, we report serious neurologic and pulmonary manifestations of IRIS, with poor outcomes in patients with extensive and disseminated disease. Neurological disease following initiation of ART due to inflammation associated with previously subclinical *M. tuberculosis* infection in the central nervous system (CNS) is of particular concern [2]. In this study, we report 4 cases (31%) of meningitis TB-IRIS. They were receiving ATT for extensive TB disease, but had no evidence of CNS involvement at time ART initiation. Following development of meningitis, all four patients were hospitalized and received steroids, but three died. The mean CD4 cell count of three meningitis patients who died was higher as compared to the other two paradoxical TB IRIS patients who died (99 vs.34 cells/μl). The published literature on CNS TB IRIS is mostly restricted to a few case reports [14,15]. A recent prospective case series from South Africa by Pepper et al, with a larger sample size (190 cases of TB IRIS) has reported neurologic TB-IRIS in 23 (12%) cases where also meningitis was the most common manifestation, followed by tuberculoma and myeloradiculopathy. In their study, Pepper et al also describe a low (70%) six month survival for neurologic TB-IRIS cases [16].

Working under program conditions, our study was limited by non-availability of plasma viral loads. Also the sample size was inadequate to define risk factors for TB IRIS or draw statistical inferences in general. However it is a precise retrospective review of data, especially outcome data, from a referral TB hospital which cares for TB patients referred from all over north India.

In conclusion, similar to other regions of the world, paradoxical TB-IRIS is a frequent problem during concomitant ATT and ART in HIV-TB co infected patients from northern India and mostly occurs during the initial weeks of ART. Meningitis is a potentially life threatening manifestation of TB-IRIS. Future studies in areas with high prevalence of *M. tuberculosis* infection should therefore address the issue of how patients at risk of developing paradoxical CNS TB-IRIS might be identified.

## Competing interests

The author(s) declare that they have no competing interests.

## **Authors' contributions**

UA was involved in conception and design of the study, interpretation of data and drafting the manuscript. AK was involved in conception of the manuscript, acquisition of data and revising the manuscript for scientific content. DB was involved in interpretation of data and revising the manuscript for scientific content. PP was involved in drafting the manuscript and interpretation of data. MAF was involved in drafting the manuscript and contributed to the scientific content of the paper. All authors read and approved the final manuscript.
